# A Sparse Representation Based Method to Classify Pulmonary Patterns of Diffuse Lung Diseases

**DOI:** 10.1155/2015/567932

**Published:** 2015-03-03

**Authors:** Wei Zhao, Rui Xu, Yasushi Hirano, Rie Tachibana, Shoji Kido

**Affiliations:** ^1^Medical Engineering Science, Graduate School of Medicine, Yamaguchi University, Ube 755-8611, Japan; ^2^Global Innovation Research Organization, Ritsumeikan University, Kusatsu 525-8577, Japan; ^3^National Institute of Technology, Oshima College, Oshima 742-2193, Japan

## Abstract

We applied and optimized the sparse representation (SR) approaches in the computer-aided diagnosis (CAD) to classify normal tissues and five kinds of diffuse lung disease (DLD) patterns: consolidation, ground-glass opacity, honeycombing, emphysema, and nodule. By using the K-SVD which is based on the singular value decomposition (SVD) and orthogonal matching pursuit (OMP), it can achieve a satisfied recognition rate, but too much time was spent in the experiment. To reduce the runtime of the method, the K-Means algorithm was substituted for the K-SVD, and the OMP was simplified by searching the desired atoms at one time (OMP_1_). We proposed three SR based methods for evaluation: SR1 (K-SVD+OMP), SR2 (K-Means+OMP), and SR3 (K-Means+OMP_1_). 1161 volumes of interest (VOIs) were used to optimize the parameters and train each method, and 1049 VOIs were adopted to evaluate the performances of the methods. The SR based methods were powerful to recognize the DLD patterns (SR1: 96.1%, SR2: 95.6%, SR3: 96.4%) and significantly better than the baseline methods. Furthermore, when the K-Means and OMP_1_ were applied, the runtime of the SR based methods can be reduced by 98.2% and 55.2%, respectively. Therefore, we thought that the method using the K-Means and OMP_1_ (SR3) was efficient for the CAD of the DLDs.

## 1. Introduction

Diffuse lung diseases (DLDs) refer to a series of abnormalities that spread out in large areas of the lungs [1]. With the development of the medical imaging technology, at present the high-resolution computed tomography (HRCT) is thought to be the best tool for the diagnosis of the DLDs, because the pulmonary patterns can be accurately analyzed on the HRCT images [2–4]. However, the interpretation of the DLD patterns mainly depends on the radiologists' individual experiences. It is reported that the agreements between the radiologists' first choices were only moderate [5]. So, the subjective differences between the radiologists may lead to the misdiagnosis. Furthermore, the HRCT produces large numbers of axial slices in each scan, which is a big burden for the radiologists. Considering the above reasons, a computer-aided diagnosis (CAD) method is required to provide the radiologists with a “second opinion” for the diagnosis of the DLDs [6–8].

In the past ten years, researchers have proposed several CAD systems to classify the DLD patterns, and most of the conventional methods aim to develop the discriminative features for the classification. For example, Park et al. adopted the statistical moments of the histograms and gray-level run-length matrices (GLRLM) to represent the textural information of the pulmonary patterns [9]. Wang et al. thought that the GLRLM could be partitioned into four areas with clear physical meaning, which can be used to measure the pulmonary textures [10]. Although the features based on the textural information have an excellent performance on the classification of the DLD patterns, these features are still difficult to recognize the pulmonary patterns with inhomogeneous textures. Therefore, researchers have combined the texture-based measures with the geometrical information to design the features with higher discriminative power. In the study [11], the pulmonary patterns were determined by six kinds of physical features, three based on the CT values (mean and standard deviation of CT values, air density components) and three based on the geometrical information (nodular components, line components, and multilocular components). Uppaluri et al. adopted the texture features and geometric fractal dimension (GFD) to classify the pulmonary patterns, where the GFD was used to measure the roughness of textures [12]. In the work [13], the measures based on the histogram, gradient, gray-level cooccurrence matrix (GLCM), and GLRLM were used for texture analysis, and the measures based on the top-hat transformation and clusters of low attenuation areas were used to analyze the shape information. Besides, the local binary pattern (LBP) was employed to quantitatively measure the normal tissues and two subtypes of the emphysema [14].

In this paper, the sparse representation approaches were introduced to recognize the DLD patterns. The main idea of the sparse representation is to approximate the example by a weighted linear combination of a small number of key features (atoms), which are selected from an overcomplete dictionary. It is thought that the sparse representation can improve the performance of the image classification [15–17]. Firstly, the images could be treated as a distribution of a set of representative features, so the sparse representation can encode the semantic information of the images. Secondly, the number of atoms in the dictionary is greater than the dimensionality of the input examples, which means that the approximation of the example is not unique. So, it can find a relative better approximation among the various combinations of atoms. Thirdly, the sparse representation is shown to be robust in the presence of the noise. Due to these advantages, the sparse representation approaches have been applied in the CAD recently. For example, Liu et al. developed a sparse representation based method to detect the colon polyp and lung nodule [18]. Vo and Sowmya trained discriminative dictionaries to classify four kinds of the pulmonary patterns [19]. In the work [20], the dictionary of the texton was learned and used to recognize the normal tissues and three subtypes of the emphysema.

**Figure 1:**
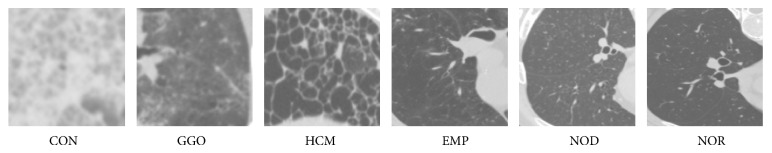
Images of six kinds of pulmonary patterns: consolidation (CON), ground-glass opacity (GGO), honeycombing (HCM), emphysema (EMP), nodule (NOD), and normal tissues (NOR).

In this work, by adopting the two of the most popular algorithms, the singular value decomposition (SVD) based K-SVD algorithm [21] and orthogonal matching pursuit (OMP), we proposed a sparse representation based method to classify the normal tissues (NOR) and five kinds of the DLD patterns, including the consolidation (CON), ground-glass opacity (GGO), honeycombing (HCM), emphysema (EMP), and nodule (NOD).  Figure 1 gives the images of the six kinds of the pulmonary patterns. According to our knowledge, there is no work aimed at applying the sparse representation approaches to analyze these six kinds of the pulmonary patterns. The proposed method using the K-SVD and OMP achieved a high classification accuracy (greater than 95%) in the experiments, which was thought to be of great potential by the radiologists. However, the runtime of this method was relatively long. Therefore, we also tried to reduce the runtime of the sparse representation based method. Considering that the operation of the K-SVD and OMP spent the most time on the training and testing, respectively, we employed the K-Means to replace the K-SVD and used a simple version of the OMP which was named OMP_1_ in the paper. Experimental results show that the replacement of the K-SVD and OMP by the K-Means and OMP_1_ can reduce the runtime of the method while keeping the classification accuracy.

There are two major differences against a preliminary version of this work [23]. Firstly, we not only adopted the sparse representation approaches for the classification but also optimized the dictionary learning and sparse coding in this work. Secondly, we changed the experimental data to make the number of training and testing samples approximately equal. This paper is organized as follows. In Section 2, we describe the proposed methods. The experimental results are given and discussed in Section 3. Finally, we conclude the paper in Section 4.

## 2. Proposed Methods

**Figure 2:**
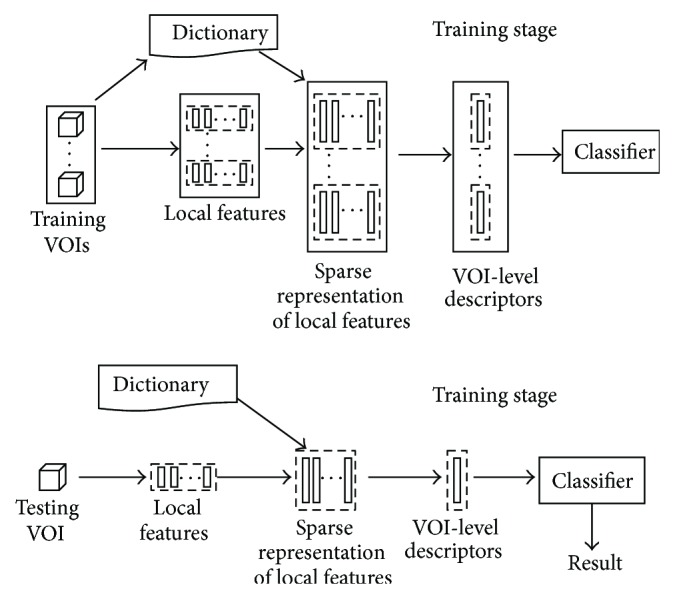
The framework of our methods.

In this research, we adopted and optimized the sparse representation approaches to classify the normal tissues and five kinds of the DLD patterns on HRCT images.  Figure 2 gives the framework of our methods. In the training stage, firstly huge numbers of local features were extracted from the training volumes of interest (VOIs) and used to train an overcomplete dictionary. Secondly, the sparse representation of the local features was calculated according to the given dictionary, and the VOI-level descriptors of the training VOIs were generated by the procedure named spatial pooling. Finally, the descriptors were used to train a support vector machine (SVM) classifier. In the testing stage, after extracting the local features on the testing VOI, the learned dictionary was adopted to calculate the sparse representation of the local features, and then the VOI-level descriptor was generated. At last, the descriptor was fed into the trained classifier and the result was given. In order to easily understand the paper, we introduce the sparse representation and its optimization at first, and then we describe the other parts of our methods.

### 2.1. Sparse Representation

Let *m* examples and the normalized overcomplete dictionary be **y**_*i*_ ∈ ℝ^*n*^, *i* = 1,2,…, *m*, and **D** ∈ ℝ^*n*×*k*^, *n* ≪ *k*, respectively, the sparse representation of *m* examples **a**_*i*_ ∈ ℝ^*k*^can be formulated as

(1) $$\begin{array}{c} \underset{D,a}{\min}\overset{m}{\underset{i=1}{\sum }}{\left\|y_{i}-Da_{i}\right\|}_{2}^{2}\;\text{subject}\;\;\text{to}\;\;{\left\|a_{i}\right\|}_{0}\leq T, \end{array}$$

where the ‖·‖_0_ means the *l* ^0^-norm indicating the sparsity of the vector (number of nonzero entries in the vector), and *T* is the threshold of the sparsity. It could be found that the **a**_*i*_ can be thought of as the coefficients of the atoms. There were two main components in the operation: (1) training an overcomplete dictionary **D** (dictionary learning); (2) calculating the sparse representation of the input example **a** according to a given dictionary (sparse coding).

By adopting the K-SVD and OMP for the dictionary learning and sparse coding, respectively, we proposed a method that was called SR1 in the paper. The K-SVD trained the dictionary by alternatively updating the coefficients with the fixed dictionary (sparse coding stage) and then updating the dictionary with the fixed coefficients (dictionary updating stage) until the stop condition was met.

1. In the sparse coding stage, it was recommended to use the OMP, a greedy technique [21]. In the beginning, the solution support was empty and an initial residual vector was evaluated by the input example. At each iteration, the atom that had the largest inner product with the residual vector was added to the support. Then the sparse approximation of the example was calculated according to the support, and the residual was updated. These processes were repeated until the number of atoms in the support was greater than the sparsity threshold.
2. In the dictionary updating stage, the columns of the** D** (atoms of the dictionary) were updated sequentially. When the *i*th atom was being updated, the reconstruction matrix except the current atom was restricted by choosing the examples which were reconstructed by using the current atom and then decomposed by the SVD. The first left-singular vector was adopted to update the atom.

After training the dictionary by the K-SVD, the SR1 also used the OMP to calculate the sparse representation of local features, the same as the sparse coding stage in the K-SVD. There were two parameters in the sparse representation, the number of atoms and the sparsity in the approximation. We adjusted the number of atoms from 500 to 3000 with an interval of 500 and the sparsity from 2 to 14 with an interval of 2 in the experiments. We present the way of optimizing the parameters in Section 3.2.

### 2.2. Optimization of Sparse Representation

The runtime of the CAD method is also an important criterion in the clinical practice. The SR1 that uses the K-SVD and OMP (see Section 2.1) can achieve a high classification accuracy, but the runtime of the SR1 was relatively long (see Section 3.3). Considering that the dictionary learning and sparse coding spent the most time on the training and testing, respectively, another aim of our research was to optimize these two steps.

Although it might be useless in the clinical workflow, we thought that the optimization of the dictionary learning can be convenient for the developers to update the existing methods. It is reported that the K-Means algorithm can achieve a competitive performance on the natural image classification with the K-SVD when the same number of atoms was used [24]. Moreover, the K-Means could be treated as a simple version of the K-SVD. In the dictionary updating stage, the average operation and SVD were adopted to update the atoms by the K-Means and K-SVD, respectively. And in the sparse coding stage, the K-Means set the coefficient of the closest atom to 1 (the values of other coefficients were 0), while the sophisticated OMP algorithm was adopted in the K-SVD. It can be deduced that the K-Means would need a shorter runtime than the K-SVD to train the dictionary. Furthermore, the K-Means can be efficiently implemented by using the *k*-dimensional tree (*k*-d tree) technique. Due to the above reasons, we tried to adopt the K-Means as a substitute of the K-SVD to train the dictionary.

In the SR1, the OMP was adopted as the solver of the sparse coding, which iteratively calculated the sparse approximation of examples, and only one atom was added to the support at each iteration. It can be deduced that the runtime of the method may be reduced by selecting enough atoms at one time. So, this approach was named OMP_1_ in the paper. After arranging the inner products of the atoms and example in a descending order, the first sufficient numbers of atoms were treated as the solution support and then used to calculate the sparse representation of the example.  Algorithm 1 gives the OMP_1_. Although the residual error of the OMP_1_ would be larger than the OMP, the performance of this approach can be ensured under a certain sparsity constraint [25].

In order to examine the performances of the sparse representation approaches, we constructed another two sparse representation based methods: SR2 (K-Means+OMP) and SR3 (K-Means+OMP_1_) in this work. The replacement of the K-SVD by the K-Means was evaluated by comparing the SR1 and SR2 (both of the two methods adopted the OMP for the sparse coding), and the substitution of the OMP by the OMP_1_ was evaluated by comparing the SR2 and SR3 (both of the two methods adopted the K-Means for the dictionary learning).  Table 1 summarizes the three sparse representation based methods and the experiments on the evaluation of the sparse representation approaches.

### 2.3. Calculation of Local Features

It is thought that the DLD patterns can be featured by a combination of CT values and measures based on the geometrical information. In this work, we used the local features proposed in the work [26] which adopted the eigenvalues of the Hessian matrix to measure the geometrical information. The local features were calculated at each sampling point on the VOI as the following procedures. Firstly, a cubic-shape patch was constructed by sampling on the VOI whose center was located on the sampling point, and four kinds of the statistical moments were calculated on this patch: mean, standard deviation, skewness, and kurtosis. Then the eigenvalues of the Hessian matrix were calculated for each voxel within the patch. Let the eigenvalues be *λ*_1_, *λ*_2_, and *λ*_3_, *λ*_1_ ≥ *λ*_2_ ≥ *λ*_3_. We arranged the eigenvalues in the order of the position. So, three new patches were constructed whose components were *λ*_1_, *λ*_2_, and *λ*_3_ respectively, and the same moments were calculated on these three eigenvalues based patches. Finally, the moments calculated on all four patches were concentrated into a 16-dimensional vector as the feature vector. In the experiments, the step of the sampling points was set to 4 × 4 × 4. And the size of the patch was a parameter, which was adjusted from 2 × 2 × 2 to 6 × 6 × 6. The way of tuning the parameter is described in Section 3.2.

### 2.4. Spatial Pooling

The procedure of the spatial pooling was used to summarize the sparse representation of the local features over the regions into a VOI-level descriptor for each VOI. These descriptors were used as the input vectors of the classifier. We adopted one of the most popular choices, the average pooling in the work, which could be seen as an average operation of the vectors. Let **z** ∈ ℝ^*k*^ be the VOI-level descriptor, let **a** ∈ ℝ^*k*^ be the sparse representation vectors, and let {·}_*t*_ be the *t*th element of the vector. The average pooling of *m* vectors is given by

(2) $$\begin{array}{c} z_{t}=\frac{1}{m}\overset{m}{\underset{i=1}{\sum }}a_{it},\;t=1,2,\ldots ,k. \end{array}$$

### 2.5. Classification

In the research, we adopted the support vector machine (SVM) as the classifier to recognize the descriptors generated in the spatial pooling. We used a version named LIBSVM [27]. It is reported that the sparse representation based classification with the linear kernel can achieve a competitive performance and smaller computational cost than the nonlinear kernels [16]. So, we employed the LIBSVM with a linear kernel. The kernel is given by

(3) $$\begin{array}{c} K\left(x_{i},x_{j}\right)=x_{i}^{T}x_{j}, \end{array}$$

where **x**_*i*_ and **x**_*j*_ are both descriptors. Because the SVM was originally designed as the binary (two-class) classifier, the LIBSVM adopted the one-against-one technique to extend the binary SVM classifier for the multiclass tasks. There is one parameter in the classifier: soft-margin penalty *C*. The way of adjusting the parameter is described in Section 3.2.

## 3. Experiments and Results

### 3.1. Data

We obtained 117 scans from 117 subjects from Tokushima University Hospital in Japan. All HRCT scans were acquired by Toshiba Aquilion 16-row multislice CT when edge-enhanced filtering was not applied. A tube voltage of 120 kVp and current of 250 mAs were used. The resolution of scans was 512 × 512, and the in-plane resolution was about 0.6 mm. The slice thickness was 1 mm.

The VOIs were constructed according to the following procedures. (1) All scans were reviewed by a radiologist, and a maximum of three axial slices was selected from the top, middle, and bottom parts of the lungs, respectively, in each scan. Only one kind of the pulmonary pattern dominantly existed on each selected slice, and the radiologist should indicate what the dominant texture was and where it existed. (2) Another two radiologists reviewed the results of the first radiologist. Only the slices which were thought to be correct by both radiologists were selected. (3) The regions of the pulmonary patterns on the selected slices were marked by all three radiologists, respectively, and the common regions chosen by the radiologists were saved. (4) The grids with a size of 32 × 32 were overlaid on the slices, and the square-shaped patches were constructed where the regions marked by the radiologists should take more than 70% area of the patches. (6) The VOIs with a size of 32 × 32 × 32 were constructed. The patches were treated as the central-axial slice of VOIs.

### 3.2. Experimental Setting

In the experiment, we separated the VOIs into two independent sets. One set (1161 VOIs) was adopted as the training set to optimize the parameters of the methods and then train the methods with the optimal parameters. The other set (1049 VOIs) was used as the testing set to evaluate the performances of the methods. There was no cross subject between the two sets. The number of VOIs of each type of patterns for the training and testing is summarized in Table 2. All methods were operated on the server with a 2.8 GHz Intel Core i7 CPU and 24 GB RAM.

**Figure 3:**
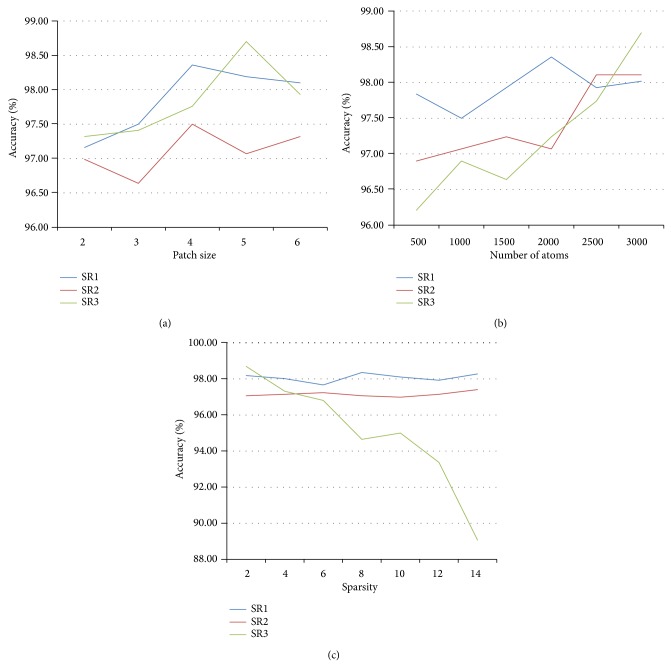
Overall accuracy of the proposed methods in the cross-validation; (a) the patch size was adjusted when the number of atoms and sparsity parameters were fixed; (b) the number of atoms was adjusted when the patch size and sparsity were fixed; (c) the sparsity was adjusted when the patch size and number of atoms were fixed.

There were four kinds of parameters in the proposed methods: the size of cube-shape patches, the number of atoms, the sparsity of the sparse representation, and the parameter related to the classifier. We tuned the values of the patch size from 2 × 2 × 2 to 6 × 6 × 6 with a step of 1 × 1 × 1, the number of atoms from 500 to 3000 with an interval of 500, and the sparsity from 2 to 14 with an interval of 2. The parameter of the SVM classifier was set to 2^−2^, 2^−1^,…, 2^11^, 2^12^. These parameters were simultaneously optimized by a 20-fold cross-validation test on the training set. The combination of the parameters which achieved the best overall accuracy in the cross-validation test was chosen as the optimal parameters. The results of the proposed methods in the cross-validation were given in Figure 3.  Figure 3(a) shows that when the patch size was nearly to the step of sampling point (4 × 4 × 4), the overall accuracy was near its maximum.  Figure 3(b) illustrates that the raising of the number of atoms can improve the performance of the methods.  Figure 3(c) shows that, with the increasing of the sparsity, the overall accuracy of the SR1 and SR2 remained, but the SR3 was decreased.

### 3.3. Three Kinds of Baseline Methods

We compared the proposed methods with three kinds of state-of-the-art published techniques, which were called SDF [11], CSE [28], and BOW [29], respectively. The parameters of the baseline methods were optimized in the same way as the proposed methods.

(1) In the work [11], the pulmonary patterns were determined by the six kinds of specially designed features. So, this method was called SDF in the paper. These six features were mean and standard deviation of CT values, air density components, nodular components, line components, and multilocular components. A three-layered artificial neural network (ANN) with back-propagation algorithm was adopted as the classifier. In the work [11], the number of hidden units in the ANN was empirically set to 10. We adjusted the number of hidden units from 5 to 30 with an interval of 5 in the experiments. Because 2D regions of interest (ROIs) were required by the SDF, we used the central slices in the axial direction of the VOIs as the ROIs in the experiments.
(2) In the work [28], the signature of the VOI was used for the classification. The signature was defined as the centroids and the weights of the clusters (number of voxels in the clusters), and the K-means algorithm was used to calculate the centroids of the clusters. In order to reduce the computational cost, the canonical signatures for each class were generated by combining and reclustering the signatures of the training data. The earth mover's distance (EMD) approach was adopted to measure the similarity between the two signatures, and the nearest neighbor (NN) was employed as the classifier. In the classification, the VOIs were recognized by comparing the signatures of the VOIs with the canonical signatures. Because the canonical signatures and earth mover's distance (EMD) were used, this method was called CSE in the paper. The CSE had only one parameter: the number of clusters. Considering that the large value was suggested to be avoided, we adjusted the number of clusters from 5 to 60 with a step of 5.
(3) The work [29] adopted a model named “bag-of-words” (also named bag-of-features) to generate the VOI-level descriptors, so this method was called BOW in the paper. The main idea of the bag-of-words was to train a code-book (dictionary) at first and then use the histograms of the words (atoms) in the code-book to represent the images. These histograms could be used as the input vectors of the classifier. In the experiments, the K-Means algorithm was adopted to cluster the local features, and the centers of the clusters were saved as the words of the code-book. The number of words was adjusted from 50 to 400 with an interval of 50. The local features adopted in the work [29] were the same as the proposed methods, so we adjusted the values of the patch size from 2 × 2 × 2 to 6 × 6 × 6 with a step of 1 × 1 × 1, the same as proposed methods. The SVM was adopted as the classifier. Considering that the *χ* ^2^ kernel achieved the best result in the work [29], the LIBSVM was employed with the *χ* ^2^ kernel. Equation ([Disp-formula EEq4]) gives the *χ* ^2^ kernel, where *α* is the parameter for the kernel and **x**_*i*_ and **x**_*j*_ are both histograms with *k*-bins:
  (4) $$\begin{array}{c} H\left(x_{i},x_{j}\right)=\exp\left[-\alpha \overset{k}{\underset{t=1}{\sum }}\frac{{\left(x_{it}-x_{jt}\right)}^{2}}{x_{it}+x_{jt}}\right]. \end{array}$$

The possible values of the soft-margin penalty and *α* were set to be 2^−2^, 2^−1^,…, 2^11^, 2^12^ and 2^−10^, 2^−9^,…, 2^1^, respectively.

### 3.4. Experimental Results

**Figure 4:**
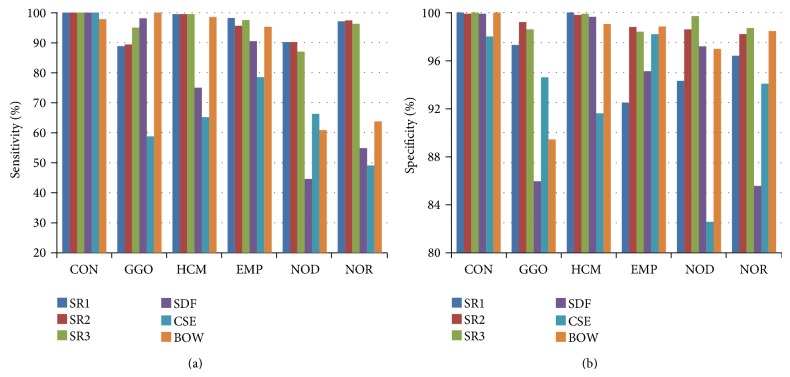
Sensitivity and specificity of each method for the pulmonary patterns with optimal parameters.

Table 3 gives the overall accuracy of each method with the optimal parameters on the testing set. The sparse representation based methods achieved better results than the baseline methods (SR1: 96.1%, SR2: 95.6%, SR3: 96.4% versus SDF: 75.8%, CSE: 65.1%, BOW: 85.5%).  Figure 4 shows that the sensitivity and specificity of the proposed methods for each pulmonary pattern were all beyond 90%, better than the baseline methods. Additionally, Table 4 shows that the *P* values of the statistical differences (calculated by the McNemar's test) for the proposed methods against the baseline methods were all smaller than 0.0001, which means that there were significant differences between the methods.

On the other hand, Table 5 compares the runtime of the proposed methods with the optimal parameters. When the K-SVD was replaced by the K-Means, the runtime of the dictionary learning can be decreased by 98.2% (SR1: 13520 s versus SR2: 241 s). When the OMP_1_ was substituted for the OMP, the average runtime of recognizing one VOI can be decreased by 55.2% (SR2: 0.29 s versus SR3: 0.13 s).

### 3.5. Discussion

Experimental results show that the sparse representation based methods had a good performance on the classification of the six kinds of the pulmonary patterns, which were thought to be of great potential for the clinical application by the radiologists. Furthermore, the replacement of the K-SVD and OMP by the K-Means and OMP_1_ can save the runtime of the method while keeping the classification accuracy. Therefore, we thought that the SR3 which adopted the K-Means and OMP_1_ was efficient in the CAD of the DLDs.

It is thought that the images could be treated as a distribution of a set of representative features, so the sparse representation can extract the important information of examples while removing the irrelevant details, which is advantageous for the classification. Although the textures of the DLD patterns on the HRCT images are complex, the sparse representation approaches are able to produce the descriptors with enough discriminating power. So, the proposed methods achieved good results in the experiments.

**Figure 5:**
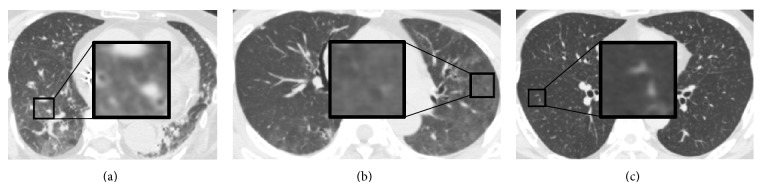
Misclassified examples by the proposed methods. (a) An example of the GGO which was misclassified to be the NOD. (b) An example of the GGO which was misclassified to be the EMP. (c) An example of the NOD which was misclassified to be the NOR.

However, the performance of the sparse representation based methods on the classification of the GGO and NOD was relatively worse. The appearance of the GGO on the HRCT image is a hazy increased in the pulmonary attenuation (“whiter” than the normal pulmonary parenchyma, but “blacker” than the soft tissues such as vessels). So, the extent of the GGO would affect the recognition. Figures 5(a) and 5(b) give two examples of the GGO which were misclassified to be NOD and EMP, respectively. Compared with the surrounding normal tissues, the abnormal extent is relatively low in the VOIs. For the NOD, the recognition would be affected by the number of nodular opacities.  Figure 5(c) shows an example of the NOD which was misclassified to be the NOR. The reason may be the few nodular opacities in the VOI.

**Figure 6:**
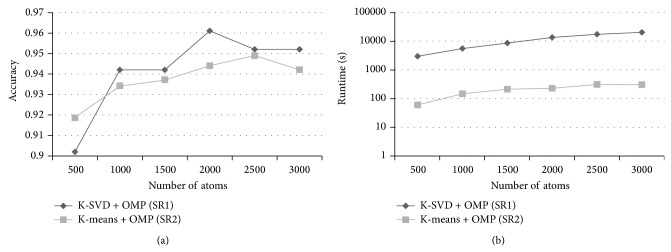
The classification accuracy and runtime of the dictionary learning of SR1 (K-SVD+OMP) and SR2 (K-Means+OMP). The patch size was 4 × 4 × 4 and the number of nonzero entries was 8. Replacement of the K-SVD by the K-Means can reduce the runtime of the method while keeping the classification accuracy.

For the CAD system, it is also important to reduce the runtime of the method while keeping the classification accuracy. Considering that the dictionary learning and sparse coding spent the most time on the training and testing, respectively, we tried to optimize these two stages. In order to reduce the runtime of the dictionary learning, we used the K-Means to train the dictionary, which could be seen as a simple version of the K-SVD.  Figure 6 compares the SR1 (using the K-SVD) and SR2 (using the K-Means). It can be found that the two methods had similar classification accuracies. Furthermore, the runtime of the dictionary learning by the K-SVD was nearly 50 times as long as the K-Means when the same number of atoms was used. It is demonstrated that the replacement of the K-SVD by the K-Means can considerably decrease the runtime and not affect the classification accuracy.

**Figure 7:**
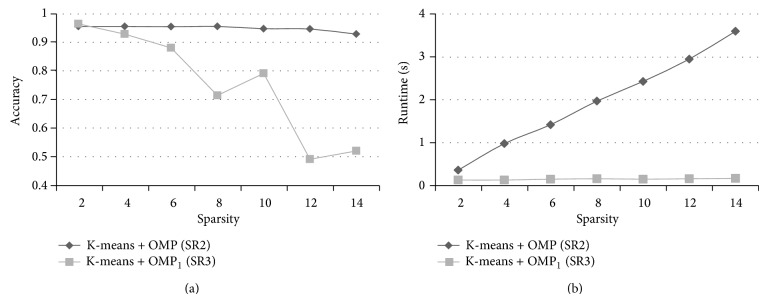
The classification accuracy and runtime of recognizing one VOI (except feature extraction) of SR2 (K-Means+OMP) and SR3 (K-Means+OMP_1_). The patch size was 3 × 3 × 3 and the number of nonzero atoms was 3000. Replacement of the OMP by the OMP_1_ can reduce the runtime of the method and achieve good performance when the value of the sparsity is small.

For the optimization of the sparse coding, although the runtime of recognizing one VOI in our experiment seemed not very long, the CAD system will be used to analyze the whole lungs of patients in the clinical practice, which can be divided into tens of thousands of VOIs. So, a small reduction of the runtime in the experiment (classify the individual VOIs) is meaningful which can lead to a remarkable decrease in the actual practice (recognize the whole lungs of patients). In order to reduce the runtime of the sparse coding, we applied a simple version of OMP, which selected the desired number of atoms at one time instead of the iterative calculation (OMP_1_).  Figure 7 compares the SR2 (using the OMP) and SR3 (using the OMP_1_) when the same parameters were used. The recognition rates of the SR3 were similar to the SR2 when the sparsity was small (2 and 4). And the SR3 spent shorter runtime than the SR2. It is demonstrated that the application of the OMP_1_ with a high sparsity can achieve a good result and reduce the runtime of the method.

We compared the proposed methods with the SDF due to the two reasons. The first one was that the SDF had been successfully applied to classify most kinds of pulmonary patterns, including normal tissues and six kinds of the DLD patterns. The second one was that the features extracted from the images were directly used as the input vectors of the classifier without a “sparse coding” step. Unfortunately, the performance of the SDF was not satisfied in the experiments. We thought that the classification may be affected by detecting the geometrical-based components (nodular, linear, and multilocular component), which is still a difficult problem in the CAD, especially for the images of the severe DLDs.

The CSE was slightly similar to the SR2 and SR3. Firstly, the K-Means algorithm was adopted in all three methods. Secondly, the signatures of the VOIs, which were used as the input vectors of the classifier, were generated according to the local features. It could seem as a “coding” step, but not the sparse coding. Therefore, the CSE was used to compare with the proposed methods. The CSE produced the worst result in the experiments. The reason for the bad performance may be that the NN classifier is naive comparing to the SVM.

**Figure 8:**
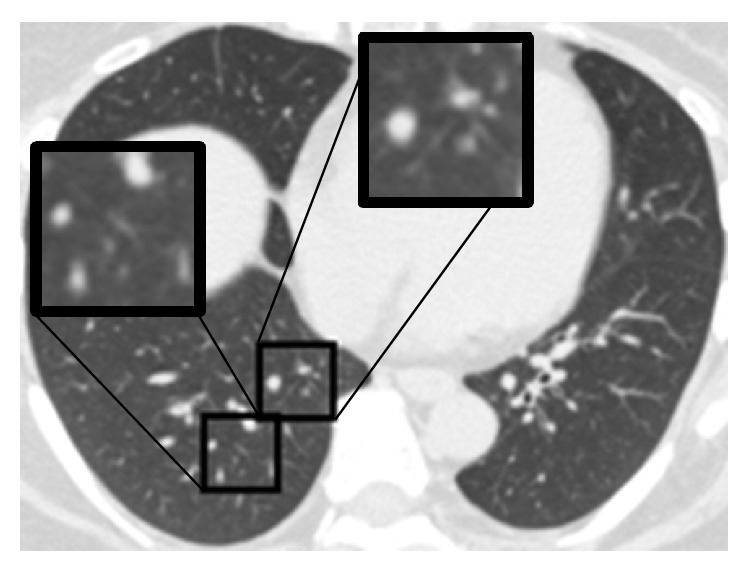
Example images of the NOR which were classified to the NOD by the BOW method.

The bag-of-words is a popular model for the image classification, and the bag-of-words based methods have achieved good results in the previous works. The bag-of-words model could be treated as a special version of the sparse representation, which was implemented with an extremely strict constraint on the sparsity. In the bag-of-words, only one atom was used to approximate the example, and the coefficient of the selected atom was fixed at 1. The work [16] thought that this constraint was too restrictive, so it would produce a large reconstruction error. For the sparse coding strategy, the sparsity constraint was relaxed by allowing a small number of atoms to describe the examples. Although more time would cost, the sparse coding approach can achieve a fine reconstruction. Therefore, it can reserve more important information of the examples, which was advantageous for the classification. On the other hand, the experimental data adopted in our experiments was different from the previous work [29]. It also would affect the classification of the BOW.  Table 6 compares the overall accuracy and runtime of the SR3 and BOW. The BOW spent little time on both the dictionary learning and recognizing. However, the SR3 achieved a significantly better overall accuracy.  Figure 8 shows two example images of the NOR which were correctly classified by the SR3 but falsely recognized as the NOD by the BOW. The reason of the misclassification may be that the appearance of these two VOIs was similar to the NOD. There were many structures with high CT values (“whiter” than the normal pulmonary parenchyma) such as vessels in the VOIs.

## 4. Conclusion

In this research, the sparse representation approaches were applied and optimized for the classification of the normal tissues and five kinds of the DLD patterns. By using the K-SVD and OMP, it achieved a satisfied recognition rate but spent too much time in the experiment. So, we tried to replace the K-SVD by the K-Means and substitute the OMP by a simple version of the OMP, which selected a sufficient number of atoms at one time (OMP_1_). Experimental results showed that the performances of the sparse representation based methods were significantly better than the baseline methods (SR1: 96.1%, SR2: 95.6%, and SR3: 96.4% versus SDF: 75.8%, CSE: 65.1%, and BOW: 85.5%). Furthermore, when the K-SVD was replaced by the K-Means, the runtime of the dictionary learning was reduced by 98.2% (SR1: 13520 s versus SR2: 241 s). And when the OMP_1_ was substituted for the OMP, the average runtime of recognizing one VOI was decreased by 55.2% (SR2: 0.29 s versus SR3: 0.13 s). Therefore, we concluded that the method using the K-Means and OMP_1_ (SR3) was efficient for the CAD of the DLDs. We will apply the SR3 in the clinical practice in future research.

## Figures and Tables

**Algorithm 1:**
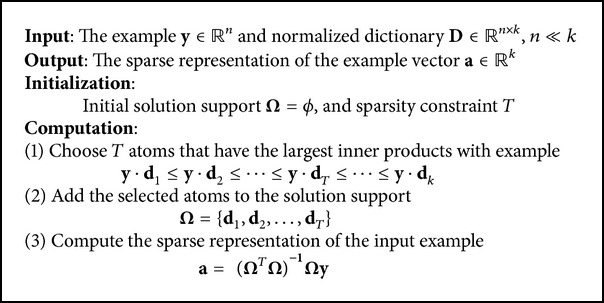
The OMP_1_ algorithm.

**Table:** (a) Proposed methods

Method	Dictionary learning	Sparse coding
SR1	*K*-SVD	OMP
SR2	*K*-Means	OMP
SR3	*K*-Means	OMP_1_

**Table:** (b) Experiments on the evaluation of sparse representation approaches

Evaluation	Comparison of methods
*K*-SVD versus *K*-Means	SR1 and SR2
OMP versus OMP_1_	SR2 and SR3

**Table 2:** Number of VOIs in the training and testing set.

	CON	GGO	HCM	EMP	NOD	NOR	Total
Training set	49	170	221	323	113	285	1161
Testing set	45	160	204	275	92	273	1049

**Table 3:** Overall accuracy of each method with optimal parameters.

Method	Overall accuracy	Optimal parameter
SR1	96.1%	Patch size: 4 × 4 × 4, number of atoms: 2000, sparsity: 8, *C*: 0.5
SR2	95.6%	Patch size: 5 × 5 × 5, number of atoms: 2000, sparsity: 2, *C*: 0.25
SR3	96.4%	Patch size: 3 × 3 × 3, number of atoms: 3000, sparsity: 2, *C*: 16
SDF	75.8%	Number of hidden units: 10
CSE	65.1%	Number of clusters: 25
BOW	85.5%	Patch size: 3 × 3 × 3, number of atoms: 300, *g*: 1.0, *C*: 4.0

**Table 4:** *P* value of statistical difference between pairs of methods.

	SR1	SR2	SR3	SDF	CSE	BOW
SR1	—	0.57	0.75	<0.0001	<0.0001	<0.0001
SR2	0.57	—	0.31	<0.0001	<0.0001	<0.0001
SR3	0.75	0.31	—	<0.0001	<0.0001	<0.0001
SDF	<0.0001	<0.0001	<0.0001	—	<0.0001	<0.0001
CSE	<0.0001	<0.0001	<0.0001	<0.0001	—	<0.0001
BOW	<0.0001	<0.0001	<0.0001	<0.0001	<0.0001	—

**Table 5:** Runtime of proposed methods with the optimal parameters.

Methods	Time of dictionary learning	Time of recognizing one VOI
SR1	13520 s (*K*-SVD)	1.27 s (OMP)
SR2	241 s (*K*-Means)	0.29 s (OMP)
SR3	350 s (*K*-Means)	0.13 s (OMP_1_)

**Table 6:** Comparison of SR3 and BOW.

	Overall accuracy	Time of dictionary learning	Time of recognizing one VOI
SR3	96.4%	350 s (*K*-Means)	0.13 s
BOW	85.5%	70 s (*K*-Means)	0.013 s
